# Effect of Temperature during Drying and Storage of Dried Figs on Growth, Gene Expression and Aflatoxin Production

**DOI:** 10.3390/toxins13020134

**Published:** 2021-02-11

**Authors:** Ana Isabel Galván, Alicia Rodríguez, Alberto Martín, Manuel Joaquín Serradilla, Ana Martínez-Dorado, María de Guía Córdoba

**Affiliations:** 1 Junta de Extremadura, Finca La Orden-Valdesequera Research Centre (CICYTEX), Horticulture, 06187 Guadajira, Spain; anaisabel.galvan@juntaex.es; 2 Food Quality and Microbiology, School of Agricultural Engineering, University of Extremadura, Avda. de Adolfo Suárez, s/n, 06007 Badajoz, Spain; amartin@unex.es (A.M.); anamd@unex.es (A.M.-D.); mdeguia@unex.es (M.d.G.C.); 3 University Institute for the Research in Agrifood Resources (INURA), University of Extremadura, Avda. de la Investigación s/n, 06006 Badajoz, Spain; 4 Junta de Extremadura, Agri-Food Technological Institute of Extremadura (INTAEX-CICYTEX), Department of Postharvest Science Avda, 06007 Badajoz, Spain; manuel.serradilla@juntaex.es

**Keywords:** mycotoxin, toxigenic moulds, food safety, figs

## Abstract

Dried fig is susceptible to infection by *Aspergillus flavus*, the major producer of the carcinogenic mycotoxins. This fruit may be contaminated by the fungus throughout the entire chain production, especially during natural sun-drying, post-harvest, industrial processing, storage, and fruit retailing. Correct management of such critical stages is necessary to prevent mould growth and mycotoxin accumulation, with temperature being one of the main factors associated with these problems. The effect of different temperatures (5, 16, 25, 30, and 37 °C) related to dried-fig processing on growth, one of the regulatory genes of aflatoxin pathway (*aflR*) and mycotoxin production by *A. flavus*, was assessed. Firstly, growth and aflatoxin production of 11 *A. flavus* strains were checked before selecting two strains (M30 and M144) for in-depth studies. Findings showed that there were enormous differences in aflatoxin amounts and related-gene expression between the two selected strains. Based on the results, mild temperatures, and changes in temperature during drying and storage of dried figs should be avoided. Drying should be conducted at temperatures >30 °C and close to 37 °C, while industry processing, storage, and retailing of dried figs are advisable to perform at refrigeration temperatures (<10 °C) to avoid mycotoxin production.

## 1. Introduction

The fig tree originates from the Middle East where it has been cultivated for millennia, probably because of well adaptation to high temperatures and low water regimes, so it has traditionally been cultivated in marginal soils under rain-fed conditions. Its fruit, the common fig (*Ficus carica* L.), is a typical species of the tropic and subtropic areas, being one of the most important agricultural products in the Middle East and Mediterranean region [1]. Fig is a seasonal fruit that can be harvested twice a year, either during the spring and summer season or in the early and late summer, depending on the cultivar [2,3]. Both fresh and dried figs are extensively consumed worldwide due to their organoleptic characteristics, important nutritional value, and natural sweetness [4]. In addition, in the last decade, production of fresh and dried figs has increased by 44% [5]. However, the high perishability of fresh fruit extremely limits the increase of area and production of this crop in the Mediterranean basin and further exportation to third countries. For this reason, the production of dried fig has been dramatically rising during the last years [5], since drying is a potential agricultural preservation technique, regardless of geographical and other challenges. Drying has proven to be a reliable preservation method for figs, in terms of technical feasibility and nutritional quality [6]. However, when temperature and duration of drying are not extremely controlled, as occurs in natural sun-drying, the hygienic-sanitary quality of figs may be affected.

Natural sun-drying has been practiced widely in tropical and subtropical countries since ancient times [7], with the main objective of ensuring the conservation of figs and extending their shelf life [8]. Apart from inconveniences caused by the uncontrolled temperature and time, the absence of meshes implies drying of figs on the ground, which in turn can lead to their infection by filamentous fungi [9]. The most predominant toxigenic fungi in dried figs are *Aspergillus* section *Nigri*, *Aspergillus* section *Flavi, Fusarium* spp., and *Penicillium* species [10,11,12]. Recently, some reports have also informed about the presence of *Alternaria* spp. in dried figs [13,14]. Some of these filamentous fungi may produce mycotoxins when the environmental factors, especially temperature and water activity (a_w_), are propitious [15,16,17]. In addition, other critical stages of dried fig processing to take into account are storage, and even during fruit retailing, since when figs are at this phase they are also susceptible to fungal colonisation and further mycotoxin production [11,18].

There are various mycotoxins found in figs including ochratoxin A (OTA), alternariol (AOH), tenuazonic acid (TeA), fumonisin B_1_, and aflatoxins [11,13,14,19,20,21]. Aflatoxins are the most important and with the highest prevalence found in figs. These mycotoxins have been found in dried figs from Turkey [11,19], Cyprus [22], and China [21]. Among the aflatoxins, aflatoxin B_1_ is recognized as one of the most potent carcinogens in foods and has been classed by the International Agency of Research for Cancer (IARC) in group 1A [23]. Due to the high toxicity of the aflatoxins and its high incidence in dried figs, the European Union has established maximum limits for aflatoxin contamination in this product at 6 μg/kg AFB_1_ and 10 μg/kg total aflatoxins (sum of AFB_1_, AFB_2_, AFG_1_, AFG_2_) [24].

In spite of these precedents, no investigations have yet been conducted about the ecophysiology of *A. flavus*, mould species producer of aflatoxins, in figs [7,25,26], under different environmental conditions occurring during fig processing. For this reason, this study is of great interest in order to investigate the capacity of *A. flavus* to grow and produce aflatoxins in a dry fig-based (DFB) medium from both phenotypic and genotypic points of view. These kinds of studies could pave the way to understand changes in the ecological status during the fig drying to comprehend the environmental conditions which favour the growth of *A. flavus* and aflatoxin production. Thus, the objective of this study was to evaluate the effect of temperature related to fig processing on growth, one of the regulatory genes of aflatoxin pathway (*aflR*) and mycotoxin production of *A. flavus* on a DFB agar at 0.96 a_w_.

## 2. Results

### 2.1. Selection of Two Aflatoxigenic Strains: Initial Screening

Initial experiments were performed using eleven *A. flavus* strains (M30, M42, M43, M55, M93, M111, M112, M115, M116, M144, and M148) to evaluate differences and similarities in their growth, lag time, and mycotoxin production capacity. For this, the *A. flavus* strains were inoculated on DFB agar 0.96 a_w_ and incubated at 25 °C for 7 days.

**Figure 1:**
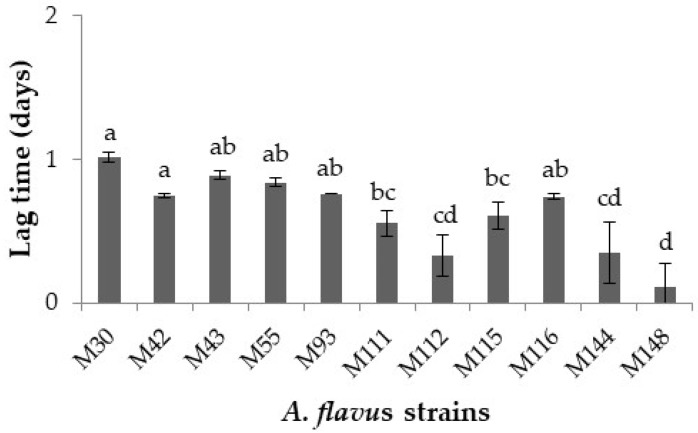
Lag time prior to growth (days) of the 11 *Aspergillus flavus* strains over the 7 day incubation period. Different letters indicate significant differences (*p* ≤ 0.05).

Figure 1 shows the combined effect of temperature, a_w_, and nutritional composition of the DFB agar on lag times prior to growth of the *A. flavus* strains tested. The lag times fluctuated between 0.11 (*A. flavus* M148) and 1.01 (*A. flavus* M30) days. Although it may appear that they were quite similar, some significant intra-strain differences (*p* ≤ 0.05) were found.

**Figure 2:**
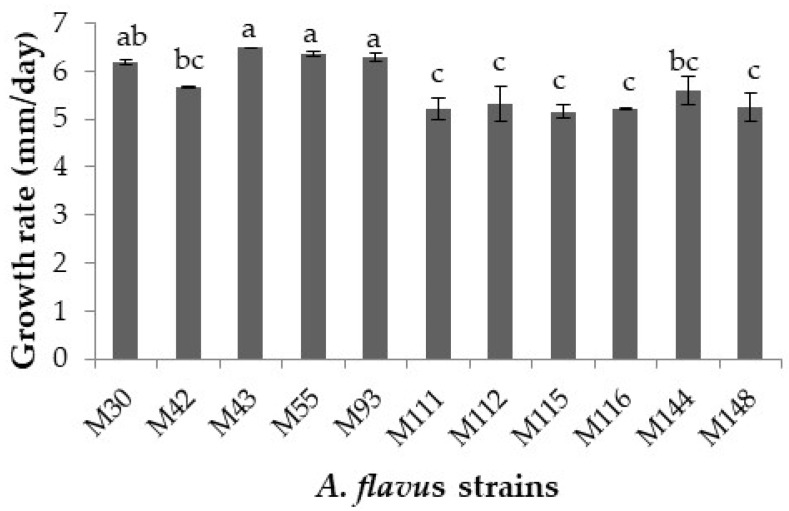
Growth rate (mm/day) of the 11 *Aspergillus flavus* strains over the 7 day incubation period. Different letters indicate significant differences (*p* ≤ 0.05).

Regarding the mean growth rates of the strains of *A. flavus*, they are displayed in Figure 2. Growth rates ranged from 5.15 (M115) to 6.49 (M43) mm radius/day. The strains M43, M55, and M93 grew faster than the remaining *A. flavus* strains checked, excluding the strain M30 (*p* ≤ 0.05). The strains M111, M112, M115, M116, and M148 showed the slowest growth of the strains evaluated.

**Figure 3:**
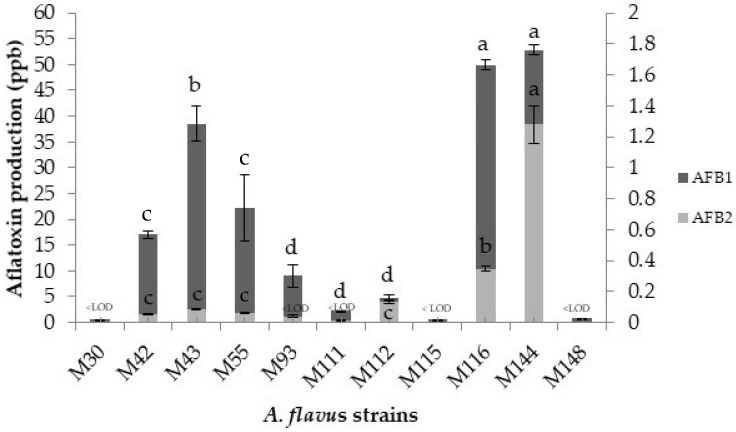
Aflatoxin production (ppb) of the 11 *Aspergillus* *flavus* strains over the 7 day incubation period. Different letters indicate significant differences for the same aflatoxins (*p* ≤ 0.05). * LOD means Limit of Detection.

With respect to aflatoxin production by the *A. flavus* strains at the specific environmental and nutritional conditions evaluated, higher intra-strain differences compared to the other two parameters analysed (lag phase and growth rates) were observed. In Figure 3, it can be observed that, in general, all the strains produced much higher amounts of aflatoxin B_1_ than aflatoxin B_2_; even in three of the strains, no aflatoxin B_2_ production was detected above the limit of detection of the technique (M30, M115, and M148). Regarding the aflatoxin B_1_, the strains M30, M115, and M148 produced aflatoxin B_1_ quantities lower than 1 ppb. Three other strains (M93, M111, and M112) synthesised this mycotoxin at levels between 2 and 9 ppb, while the remaining 5 strains produced aflatoxin B_1_ quantities higher than 17 ppb, the strains M144 and M116 being the highest producers of this mycotoxin. With regard to aflatoxin B_2_, the maximum amount synthesised was 1.28 ppb by the strain M144. All the other *A. flavus* produced this mycotoxin at levels below 1 ppb.

Based on the results obtained, the strains *A. flavus* M30 and *A. flavus* M144 were selected to carry out a more detailed study to study the lag time, growth, aflatoxin contamination, and related gene expression of *A. flavus* in relation to ecophysiological parameters linked to dried-fig production. These two strains were selected based on their lowest and highest aflatoxin production of the 11 strains isolated from dried figs.

### 2.2. Effect of Temperature on Lag Times, Growth Rates, Mycotoxin Production and Aflatoxin-Related Gene Expression

#### 2.2.1. Lag Times Prior to Growth

**Figure 4:**
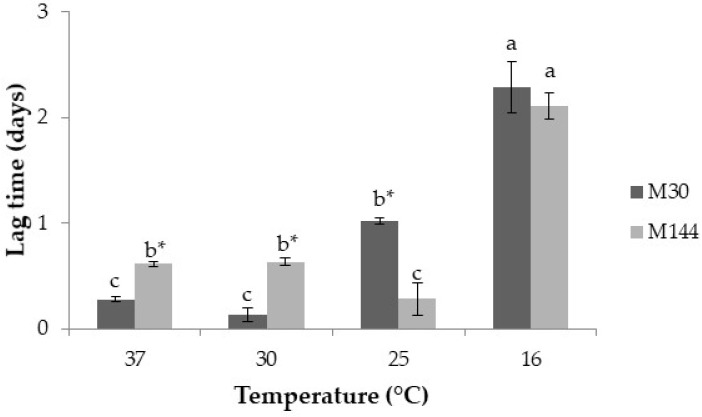
Lag time prior to growth (days) of the *Aspergillus flavus* M144 and *A. flavus* M30 at the different temperatures studied over the 12 days incubation period. Different letters indicate significant differences at the different temperatures for the same strain (*p* ≤ 0.05). Asterisk (*) means significant differences between both strains at the same temperature (*p* ≤ 0.05).

Figure 4 shows the effect of temperature related to the dried-fig processing on lag times prior to growth for both strains of *A. flavus* (M30 and M144). For both strains, no growth occurred at 5 °C. At the warmer temperatures tested (37 and 30 °C), *A. flavus* M30 had shorter lag phases than *A. flavus* M144, while at 25 °C, the latter showed the shortest lag time. At the lowest temperature evaluated, no differences were found between both strains. In addition, for both strains, the length of the lag phase rose substantially as temperature decreased.

#### 2.2.2. Growth

**Figure 5:**
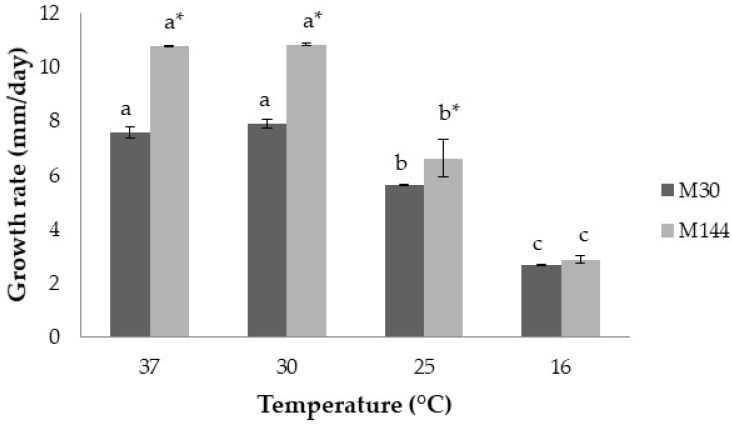
Growth rate (mm/day) of the *Aspergillus flavus* M144 and *A. flavus* M30 at the different temperatures studied over the 12 days incubation period. Different letters indicate significant differences at the different temperatures for the same strain (*p* ≤ 0.05). Asterisk (*) means significant differences between both strains at the same temperature (*p* ≤ 0.05).

The influence of temperature on the growth of both strains of *A. flavus* is shown in Figure 5. *A. flavus* M144 grew faster than *A. flavus* M30 in most of the conditions tested (*p* ≤ 0.05), although no significant differences were found at 16 °C (*p* > 0.05). Optimum growth rates (≈11 and 8 mm/day for *A. flavus* M144, and *A. flavus* M30, respectively) were observed at 30 and 37 °C in both strains. Besides, no intra-strain differences were encountered at 30 and 37 °C. Furthermore, the growth of both strains declined as the temperature fell down.

#### 2.2.3. Aflatoxin Production

Table 1 shows the effect of temperature on AFB_1_ and AFB_2_ production by *A. flavus* M144 and *A. flavus* M30 after 3, 5, 7, and 12 days of incubation. Neither AFB_1_ nor AFB_2_ was produced by the strain *A. flavus* M30 at the conditions and times evaluated (<LOD: Limit of Detection). Regarding the strain *A. flavus* M144, it produced much higher quantities of AFB_1_ than AFB_2_ in all the conditions tested. However, it should be emphasized that, in spite of the fact that there were differences regarding both mycotoxins produced by the strain *A. flavus* M144, the tendency was quite similar. Thus, maximum AFB_1_ and AFB_2_ production were detected at 25 °C at the four days tested; being detected in general higher amounts as the incubation period increased. At the remaining temperatures studied, AFB_1_ and AFB_2_ production was observed at 16 °C by day 12 of incubation.

#### 2.2.4. Gene Expression Studies

**Figure 6:**
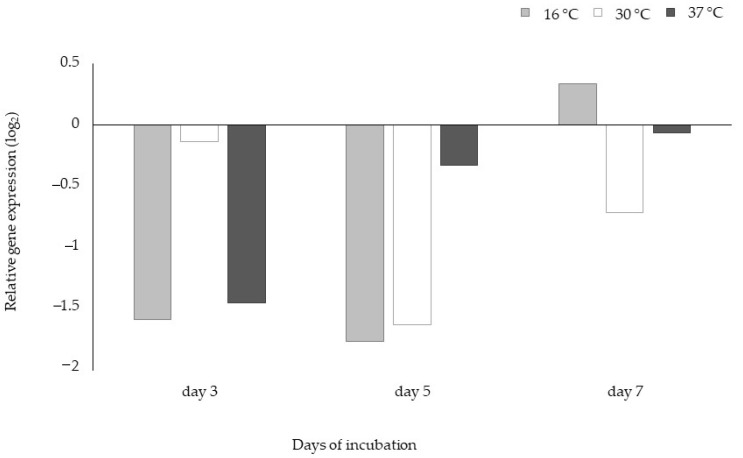
Effect of the temperature on the expression of the *aflR* by *Aspergillus flavus* M144 at different incubation times. The calibrator corresponded to *A. flavus* when grown at 25 °C.

The effect of incubation days on *aflR* gene expression of *A. flavus* M144 at different temperatures is shown in Figure 6. The incubation temperature of 25 °C was used as a calibrator in this study. As shown in Figure 6, in the case of the expression of *aflR* gene is inhibited in most cases at temperatures of 16, 30, and 37 °C and all incubation times evaluated, with the exception of day 7 at 16 °C. In the case of the strain M30, no changes in the expression of the tested regulatory gene at the different temperatures evaluated regarding the control occurred (data not shown).

## 3. Discussion

This is the first study to examine the impact of temperature on growth, *aflR* gene expression, and aflatoxin production by *A. flavus* in a dry fig-based matrix. This species has been encountered in dried figs and can cause accumulation of aflatoxins in this commodity [7,9,11]. Özlüoymak [27] has reported that the critical period for *A. flavus* for starting to grow is when the ripening of the figs is occurring on the tree and it continues during the over-ripening period. Besides, environmental conditions occurring during processing of dried fig and storage when dried figs are launched into the market where temperatures are rarely controlled also favored growth and development of *A. flavus*. Once this species colonizes fig, it may synthesise aflatoxins, both on the surface and the inner part of the fig without damaging the skin [28]. It is thus important to understand the ecological conditions for growth, aflatoxin-related gene expression and aflatoxin production by this species in this matrix. This can be useful for targeting control strategies to minimize mycotoxin contamination within the HACCP framework in the dried fig industry.

At first, the growth behavior and mycotoxin synthesis ability of 11 *A. flavus* strains isolated from figs at a fixed temperature (25 °C) in a DFB agar were screened in order to further select 2 strains based on the initial results obtained for in-depth ecophysiological studies. The initial experiment results showed that there were relatively few interspecies significant differences on lag phase and growth, whilst this was not true for aflatoxin production. Regarding the two parameters related to mould growth, the lag phases ranged between 0.11 and 1 days, while mean growth rates varied from 5.15 to 6.49 mm/day. These values indicate that *A. flavus* starts to grow immediately on a DFB medium and the nutritional composition of this medium based on fig favors the rapid growth of this toxigenic species. This is supported by the comparison of the results of the present study with previous reports informing about the lag phases and growth of *A. flavus* in different food-based model systems. For instance, Peromingo et al. [29] demonstrated that two strains of *A. flavus* had little differences on both lag phase prior to growth and growth when growing on two dry-cured meat product-based medium at 25 °C. Casquete et al. [30] observed little differences between three strains of *A. flavus* at different a_w_ in a cheese model system. With regard to aflatoxin synthesised by the 11 *A. flavus* strains, there were higher significant differences at strain level, varying aflatoxin B_1_ amounts produced between 0.6 and 50 ppb, while for aflatoxin B_2_ they were in the range from <LOD to 1.28 ppb. Previous studies have also shown differences in aflatoxin synthesis by various *A. flavus* strains at 25 °C in different media [29,31]. Besides, in general, they produced much higher quantities of aflatoxin B_1_ than aflatoxin B_2_ in DFB agar. In this study, two *A. flavus* strains were selected based on their mycotoxin production capacity, being the strains M144 (aflatoxin-producing strain) and M30 (non-aflatoxin-producing strain) used for examining the impact of temperature on growth, aflatoxin-related gene expression and mycotoxin production by *A. flavus* in DFB agar.

Temperature represents a key environmental factor in the growth and production of aflatoxins [32,33]. For this reason, five different temperatures, which were selected due to their importance during the drying, processing, and retailing of fig fruits, were assessed. For this: 5 °C represents the advisable household and industrial storage temperature; 16, 25 and 30 °C are common minima, average and maximum temperatures during harvest stage at night, respectively, and 37 °C represents extreme temperatures that can occur in the field during the harvest of the fruits (Extremadura a southwest Spanish region in the high summer season; http://redarexplus.gobex.es/RedarexPlus, accessed on 20 December 2020). In addition, 16 and 25 °C are usual intermediate ambient temperatures utilized by both consumers and producers to store dried figs. Also, 25 °C is the usual temperature in the dried fig postharvest. Finally, 37 °C also represents the optimum condition for *A. flavus* growth [32].

When studying the influence of temperature on growth parameters of the two selected *A. flavus* strains, overall, both strains were unable to grow at 5 °C over the 12 day incubation period of our experiments. These results are consistent with several investigations that suggest that growth at a temperature below 10 °C does not occur [29,34]. Regarding the other temperatures, despite some differences found between the two strains, in general, the lag phases were shorter and mean growth rates faster as temperature increased (*p* ≤ 0.05). These results are in accordance with those published by Mohale et al. [35], who investigated the growth of toxigenic and atoxigenic *A. flavus* strains at 20, 25 and 30 °C, and also with those published by Schmidt-Heydt et al. [32], who showed that the growth optimum for *A. flavus* was at 37 °C. Pitt and Miscamble [36] reported that the optimum temperature for *A. flavus* growth was 25 °C in the range from 0.96 to 0.98 a_w_, 30 °C at 0.985 a_w_ and 37 °C at 0.96 a_w_. Other previous studies on *A. flavus* growth on groundnuts suggest a_w_ optima of 0.94 a_w_ at 34 °C [37]. Abdel-Hadi et al. [16] found that optimum growth of *A. flavus* was 0.99 a_w_ and 35 °C on conducive YES medium. Surprisingly, the strain M144 (aflatoxin-producing strain) initiated its growth slightly later than the other strain tested (M30, non-aflatoxin-producing strain), but its mean growth rate was more rapid at temperatures warmer than 25 °C. Probably, in the case of the strain M144, the synthesis of aflatoxins itself would have been of great help for its adaptation and colonisation of the DFB agar. This phenomenon has been described before [38,39].

Findings from aflatoxins produced by the two strains of interest showed enormous differences at strain and species levels. The aflatoxin produced by both *A. flavus* at 5 °C was not tested since growth was not observed. The strain M30 did not produce aflatoxins either in temperature or incubation day evaluated. The strain M144 produced both aflatoxin B_1_ and aflatoxin B_2_, but the quantities produced of the most carcinogenic were much higher (*p* ≤ 0.05). As expected, the largest aflatoxin B_1_ and quantities detected were at 25 °C (*p* ≤ 0.05); however, also important amounts of such toxin would have been contemplated at 30 °C according to the results reported by Schmidt-Heydt et al. [32], who evaluated the effect of a wide range of a_w_ and temperatures on *A. flavus*, although this was not observed in this work. At the warmest temperature checked (37 °C), no aflatoxin production was observed, while at 16 °C, at the end of the incubation time the strain synthesised aflatoxin B_1_ amounts > 10 ppb. These results correlate with those published with Schmidt-Heydt et al. [32]. In the same manner, aflatoxin B_2_ was more produced by this strain at 25 °C and later at 16 °C. So, it seems that the temperature enormously affects aflatoxin production by *A. flavus* independently of the substrate where the mould grows. In general, it should be emphasised that the amounts of aflatoxin found in the DFB agar are higher than those found in other culture media, food-based model systems, or food matrices [29,30,31]. The explanation may be that the preferred carbon sources for aflatoxin production are sugars [40], and dried figs provide a rich source of glucose and fructose [7]. Furthermore, the temperature of 25 °C and a 0.96 a_w_ are optimal for the growth *of A. flavus* [41].

Regarding the assessment of the expression of the *aflR* gene of the strain M144, the major regulatory gene in the aflatoxin pathway, which activates the aflatoxin structural genes [42], it was observed that, in general, this gene expression was repressed throughout the incubation time and at any of the temperatures evaluated with respect to the calibrator (25 °C). This is in accordance with results obtained in the phenotypic mycotoxin production, where maximum amounts were found at 25 °C. These findings are reasonable since the *aflR* gene controls are well-correlated with aflatoxin production by *A. flavus* [32,43,44]. Unsurprisingly, a basal expression of the regulatory gene occurred with no differences between conditions checked in the case of the non-producing strain (*A. flavus* M30).

## 4. Conclusions

The effect of temperature during drying and storage of dried figs has a profound effect on lag times prior to growth, relative growth rates, *aflR* gene expression and aflatoxin production by strains of *A. flavus* isolated of such fruit. In general, the capacity of colonisation of the dried fig-based model system was similar to all the strains tested; however, their ability to produce aflatoxins varied between strains. Concretely, there are some important differences between the two selected *A. flavus* (M144, important producing-strain and M30, non-producing strain). Based on the results, mild temperatures and changes in temperature during drying and storage of dried figs should be avoided. Drying should be conducted at temperatures > 30 °C and close to 37 °C, while industry processing, storage, and retailing of dried figs are advisable to perform at refrigeration temperatures (<10 °C) to avoid mycotoxin production.

## 5. Material and Methods

### 5.1. Mould Strains

Eleven strains belonging to *A. flavus* previously isolated from dried figs (*Ficus carica* L.) from different geographical areas of Extremadura (a southwest region of Spain) were used in this study. Information about the isolate codes, origin, geographical area, and moisture content of the strains is shown in Table 2. Isolation of the strains was made following the protocol described by Ruiz-Moyano et al. [45]. For this, genomic DNA from the 11 moulds isolated was extracted with the quick-DNA Fungal/Bacterial Miniprep Kit (Zymo research) according to the manufacturer’s instructions. The ITS rDNA region was amplified using the primer pairs ITS1 and ITS4 described by White et al. [46]. PCR products were sequenced at the Facility of Bioscience Applied Techniques of SAIUEX (University of Extremadura, Spain) with the same primers used in the amplification steps. Sequencing was performed from both the 5′ and the 3′ ends of each PCR product. The obtained sequences were edited and assembled into a consensus sequence of the corresponding amplicon. To determine the closest known relatives of the obtained ITS rDNA sequences of the isolates, searches were performed against the NCBI nucleotide (nr/nt) database with the Basic Local Alignment Search Tool (BLAST) tool (http://blast.ncbi.nlm.nih.gov/Blast.cgi, accessed on 4 January 2021). All sequences were separately analysed and > 95% similarity was used as the criterion for species identification. The isolates were maintained by regular subculturing in Potato Dextrose Agar (PDA) at 25 °C for 7 days and then kept at 4 °C for short-term storage until required.

### 5.2. Culture Medium Preparation

DFB agar was prepared with 30 g of lyophilised dried fig which were added to 300 mL of deionised sterile water and blended with a hand mixer. The remaining deionised sterile water was added to complete 1 L and it was brought to a boil. Subsequently, 20 g of bacteriological agar (Pronadisa, Madrid, Spain) were added and mixed vigorously. The culture medium was sterilised by autoclaving at 121 °C for 20 min (103 KPa). After autoclaving, the DFB agar was shaken, and poured into 9 cm diameter Petri plates. The a_w_ of the DFB agar was measured by using a Novasina LabMaster-a_w_ meter (AG, Lachen, Switzerland).

### 5.3. Inoculum, Inoculation, and Experimental Settings

For inoculum preparation, the isolates were inoculated by spreading on PDA and incubated at 25 °C for 7 days. The spores of each mould isolate were collected using 10 mL deionised water containing 0.05% Tween 80 and rubbing the surface with a glass rod. The spore suspensions were quantified with the aid of a microscope (Olympus CX 400, Tokyo, Japan) and a Neubauer chamber before their adjustment to 10^6^ spores/mL by diluting with deionised water to be used as inoculum. The spore suspensions were maintained for long-term storage at −80 °C in glycerol solution (50% *v*/*v*). New starter cultures were used for each experiment.

Firstly, an initial screening of the mould isolates were done. For this, DFB agar was centrally inoculated with 2 μL of the inoculum of each of the 11 mould isolates and incubated at 25 °C for a period of up to 7 days. The growth assessment and aflatoxin production were tested. The two isolates which obtained the highest (*A. flavus* M144) and the lowest (*A. flavus* M30) aflatoxin production were selected to carry out detailed studies on the relationship between ecophysiological factors, growth, gene expression, and aflatoxin contamination.

Secondly, the *A. flavus* M144 and M30, selected from the initial screening experiment, were 2-point inoculated on DFB agar with 2 μL of each inoculum for growth and aflatoxin production. For gene expression studies, sterile cellophane overlays (Packaging Limited, London, UK) were placed onto DFB agar before inoculation. The agar plates were incubated at 5, 16, 25, 30, and 37 °C for up to 12 days to simulate the wide range of conditions throughout the sun-drying process, industrial processing, storage, and retailing of dried figs. The a_w_ of the medium kept constant during the experiment period. All experiments were done with three replicates per treatment and repeated once.

### 5.4. Lag Time Prior to Growth and Growth Assessment

Growth was daily recorded by measuring two right angles diameters. Data were analysed using a primary model by plotting colony diameter against time. Data plots showed, after a lag phase, a linear trend with time. The linear part of this graph (linear phase) was used to calculate growth rate (μ, mm/d) [47]. To calculate the lag times (days), the formula of the regression line was equalised to the original inoculum size (diameter, mm).

### 5.5. Gene Expression Analysis

#### 5.5.1. Sampling and Sample Preparation

For gene expression analysis, samples from strains M144 and M30 were taken at 3, 5, and 7 days of incubation. All experiments were made in triplicate.

After each incubation time, the cellophane disks containing the whole colonies were collected under sterile conditions and quickly frozen in liquid nitrogen and stored at −80 °C until RNA extraction.

#### 5.5.2. RNA Extraction

For RNA extraction, frozen mycelia were ground to fine powder in a pre-frozen mortar and pestle. Next, approximately 50 mg of frozen mycelia were weighed in a sterile Eppendorf, and the RNA extraction was carried out using the Spectrum^TM^ Plant Total RNA Kit (SigmaAldrich, St. Louis, MO, USA). The RNA concentration and purity (A_260_/A_280_ ratio) were determined spectrophotometrically using a 1.5 μL aliquot on a NanoDrop (Thermo Scientific™ *NanoDrop* 2000). Samples were diluted to a concentration of 0.1 μg/μL and treated with DNAse I (Thermo Fisher Scientific, Waltham, MA, USA) in order to remove genomic DNA. Then RNA was kept at −80 °C until reverse transcription (RT) reaction.

#### 5.5.3. RT-qPCR Reactions and Relative Quantification

RT-qPCR assays were used to amplify the *aflR* gene as target gene, and the *β-tubulin* gene as endogenous gene.

1. Primers

The primer pair aflRtaq1/aflRtaq2 previously designed from the *aflR* gene associated with the aflatoxin biosynthesis pathway [43], and the primer pair F-TubJD/R-TubJD designed from the *β-tubulin* gene [43] were used.

2. cDNA synthesis

The RT reaction was conducted by using 5 μL of total RNA (100 ng) according to the instructions of PrimeScript™ RT Reagent Kit (Takara Bio Inc., Kusatsu, Shiga, Japan). cDNA samples were stored at −20 °C for subsequent qPCR analysis.

3. Real-time PCR reactions

The real-time PCR (qPCR) reactions were performed in the 7300 Real-Time PCR System (Applied Biosystems, Foster City, CA, USA) using the SYBR Green system. Reaction mixtures were dispensed into wells of MicroAmp Optical 96-Well Reaction Plates and sealed with optical adhesive covers (Applied Biosystems). Three replicates of a RNA control sample together with a template-free negative control were also included in the runs. The reaction mixture for each gene consisted of 7.5 μL NZY qPCR Green Master Mix 2x (NZYTech, Lisbon, Portugal), 300 nM of each primer and 2.5 μL of cDNA in a final volume of 12.5 μL. PCR reaction conditions included a first step of 10 min at 95 °C, and 40 cycles of 95 °C for 15 s and 60 °C for 1 min. After the final PCR cycle, the melting curve of the PCR products was analysed according to the following protocol: slow ramp between 60 and 95 °C in 0.5 °C increments for 5 s. The value of the quantification cycle (Cq), which corresponds to the intersection between each fluorescence curve and a threshold line was automatically calculated by the 7300 Fast System Software (Applied Biosystems). Three technical repetitions were made.

4. Relative gene expression

Relative quantification of the expression of the *aflR* gene expression was calculated following the 2^−ΔΔC^_T_ method [48]. The *β-tubulin* gene was used as the endogenous control to normalise the quantification of the cDNA target added to each reaction. The calibrator corresponded to *A. flavus* when grown at 25 °C, a usual temperature in the dried fig postharvest, storage, and harvesting.

### 5.6. Mycotoxin Analysis

#### 5.6.1. Sampling and Sample Preparation

After 3, 5, 7, and 12 days of incubation, the agar plates containing the whole colonies were immediately stored at −20 °C until use. Aflatoxin content could not be determined at 5 °C since no growth of *A. flavus* occurred.

#### 5.6.2. Aflatoxin Extraction and Quantification

All solvents used for aflatoxin were HPLC grade and purchased from Thermo Fisher Scientific (Runcorn, UK). The isolation and purification of aflatoxins was conducted following the method described by Rodríguez et al. [49]. Then, the dry extracts were redissolved in 1 mL of HPLC-grace acetonitrile (Fisher Scientific) and filtered through a 0.22 PTFE membrane filter, in vials for quantification. The aflatoxin analysis was performed using an Agilent 1100 Series HPLC system (Agilent Technologies, Santa Clara, CA, USA) equipped with a FLD detector (Agilent G1321A) fitted at 360 nm and using a C18 HPLC column (250 × 4.6 mm, 5 μm particle size; Supelco, Bellefonte, PA, USA). The injection volumen was 100 μL and the flow rate was 1 mL/min. The mobile phase used for the separation contained HPLC grade water (solvent A) and HPLC grade acetonitrile (solvent B), in a gradient mode established from 15% B in the initial phase to 100% B after 30 min. Standard curves for calibration purpose were performed using standards of aflatoxin B_1_ and B_2_ acquired from Sigma-Aldrich.

### 5.7. Statistical Análisis

Data on lag phase, growth rates, *aflR* gene expression and toxin production were tested for normality using the Shapiro–Wilk test. A statistical analysis of the parameters was performed using one-way ANOVA. The differences among means values were separated by Tukey’s honestly significant difference test (*p* ≤ 0.05) in SPSS for Windows version 21.0.

## Figures and Tables

**Table 1:** Aflatoxin B_1_ and B_2_ production (ppb) of *Aspergillus flavus* M144. ^1^

Aflatoxin	Days of Incubation	37 °C	30 °C	25 °C	16 °C
B_1_	12	<LOD ^2^	0.25 ± 0.35^a3^	60.63 ± 7.70^a1^	10.15 ± 1.56^a2^
	7	<LOD	0.03 ± 0.01^c3^	58.39 ± 1.93^a1^	0.10 ± 0.07^b2^
	5	<LOD	0.12 ± 0.04^b3^	2.68 ± 0.51^b1^	0.03 ± 0.01^b2^
	3	<LOD	0.02 ± 0.01^c2^	1.26 ± 0.83^b1^	<LOD
B_2_	12	<LOD	<LOD	0.02 ± 0.01^b^	<LOD
	7	0.10 ± 0.01^a2^	0.06 ± 0.00 ^2^	0.15 ± 0.06^a1^	0.13 ± 0.01 ^1^
	5	0.02 ± 0.01^b^	<LOD	<LOD	<LOD
	3	<LOD	<LOD	<LOD	<LOD

^1^ The strain M30 did not produce detectable amounts of aflatoxin B_1_ and B_2_. ^2^ LOD: Limit of detection. Different letters along a column indicate significant differences at the different incubation times for the same temperature and for each aflatoxin (B_1_ and B_2_) (*p* ≤ 0.05). Different numbers along a row indicate significant differences at the different temperatures for the same incubation time and the same aflatoxin (*p* ≤ 0.05).

**Table 2:** Codes, geographical area, moisture content, and origin of the 11 strains of *Aspergillus flavus* used in the present study.

Isolate Code	Geographical Area	Origin ^1^	Moisture Content (%)
*A. flavus* M30	South of Extremadura	Field	16.78
*A. flavus* M42	South of Extremadura	Field	16.78
*A. flavus* M43	South of Extremadura	Field	16.39
*A. flavus* M55	South of Extremadura	Field	16.78
*A. flavus* M93	South of Extremadura	Field	20.46
*A. flavus* M111	South of Extremadura	Field	20.46
*A. flavus* M112	South of Extremadura	Field	19.01
*A. flavus* M115	South of Extremadura	Industry	27.62
*A. flavus* M116	South of Extremadura	Industry	27.62
*A. flavus* M144	North of Extremadura	Field	36.20
*A. flavus* M148	South of Extremadura	Field	16.39

^1^ Field or industry.

## Data Availability

The data presented in this study are available in the article.
